# Misperception of Oral Health among Adults in Rural Areas: A Fundamental but Neglected Issue in Primary Healthcare

**DOI:** 10.3390/ijerph15102187

**Published:** 2018-10-07

**Authors:** Mei-Yen Chen

**Affiliations:** 1 Department of Nursing, Chang Gung University of Science and Technology, Chiayi 613, Taiwan; meiyen@mail.cgust.edu.tw; 2 Department of Nursing, Chang Gung University, Taoyuan 333, Taiwan; 3 Department of Cardiology, Chang Gung Memorial Hospital, Yunlin 638, Taiwan

**Keywords:** misperception, oral health, oral hygiene, tooth loss

## Abstract

*Background:* Poor oral hygiene is associated with metabolic syndrome, systemic diseases, mortality and many chronic diseases. Misperception means a wrong or incorrect understanding or interpretation. Few studies have examined the subjective perception and objective condition of oral health among community adults in rural areas. *Methods:* This was a cross-sectional, community-based study. Participants were recruited via convenience samples from December 2015 to July 2016. One thousand six (1006) community residents participated in the project, of which 973 fulfilled the inclusion criteria. The average age was 42.8 (SD = 12.3) years, range 20–64, selected from a collaboration local hospital. *Results:* Most of the participants reported brushing their teeth every day, however, 72% reported seldom brushing their teeth after meals, 54% seldom used dental floss, 64% seldom received dental scaling, 29.5% had experienced a toothache within 6 months, and 30.5% demonstrated significant tooth loss. However, most of them perceived their oral health as good. Misperception of oral health was common, 21.5% among whose number of remaining teeth <25 under-assess their actual oral health. The more number of remaining teeth (*p* < 0.001) and regular dental check-up (*p* < 0.01) were positively associated with feeling good about their oral health. After adjusting for potential confounders, clinically significant findings indicated that number of remaining teeth (OR = 3.03, *p* < 0.001), age (OR = 0.99, *p* < 0.001), regular dental check-ups/scaling (OR = 1.85, *p* < 0.001), education (OR = 1.45, *p* < 0.05), and water consumption (OR = 1.38, *p* < 0.05) were independently associated with good perceived oral health. *Conclusions:* The findings showed that subjective self-perception of oral health was not matched with their objective oral condition. Excluding the unmodifiable factors, the clinical implications indicated that oral health promotion programs, particularly for adopting regular dental check-up, healthy diet and oral hygiene habits are urgent in rural areas.

## 1. Introduction

The World Health Organization had pointed out that oral health is essential to general health and quality of life, and optimal oral health is defined as a state of freedom from oral discomfort, periodontal disease, tooth decay, and tooth loss [1]. Misperception means a wrong or incorrect understanding or interpretation. Some studies have reported that oral disease is associated with metabolic syndrome, systemic diseases, and oral disease would increase risk of stroke, ischemic heart disease, cognitive impairment, and cardiovascular mortality [2,3,4]. Risk factors for oral diseases include unhealthy diet, tobacco use, betel nut chewing, poor oral hygiene, and social determinant [1,5,6,7,8]. Additionally, research has shown that poor oral hygiene was associated with periodontitis, which is an oral disease of microbial origin, characterized by loss of the attachment apparatus of the tooth, resulting in tooth loss and low-grade systemic inflammation [1,9]. Tooth loss frequently causes some difficulty in masticatory performance and swallowing [10]. Furthermore, poor oral health can exert a negative impact on overall quality of life, and lead to life-threatening illnesses such as heart disease, stroke, malnutrition, and pneumonia [10,11].

Many of these negative oral health effects are preventable. According to the literature on the subject, brushing the teeth twice per day, using dental floss before bedtime, adopting a healthy diet with sufficient fruit and vegetables, and receiving regular dental check-ups and scaling, all can benefit oral health and constitute fundamental content for dental health education [12,13,14]. Despite the importance of oral health, it is often neglected in primary healthcare and nursing in clinical and community settings [6,15,16,17]. Therefore, early detection and reduction of these risk factors would exert an important effect on many chronic diseases that lead to considerable national economic and health burden.

Taiwan has implemented national health insurance, covering 99% of the population for more than 20 years, and the majority of citizens are highly satisfied with the program. However, hospitals are overused for oral diseases (e.g., caries and periodontitis), exerting a burden on the limited healthcare resources [14]. Although 12 years of education has been compulsory in Taiwan for many years, and dental hygiene education is provided in all primary schools, this does not necessarily benefit adults, particularly in rural areas. Previous studies have indicated that poor oral health is often found in disadvantaged areas, around 84% of adults, 59% of elderly adults, and only 26% of rural adults with diabetes preserving 20 or more of their original teeth [2,4,14]. Japan initiated the national “8020 campaign” which involves the goal of retaining at least 20 teeth at 80 years old [1]. Research has indicated that “8020 achievers” exhibits superior bone mineral density, balance ability, grip strength, and exercise function; a higher cumulative survival rate; and greater satisfaction with life, relative to non-achievers [18].

Oral healthcare is fundamental in nursing, including that which is provided in hospital and community settings [15,16,19]. Oral healthcare has also been identified as a core component of recommended practice for reducing ventilator-associated pneumonia, as put forth by the CDC [20]. However, most nursing studies have examined oral health in elderly individuals, intensive care patients, long-term care units, and/or end-of-life patients [19,21,22,23], few studies have focused on detection of misperception of oral health status and associated factors among rural community adults. These studies offer little clinical relevance, as it is often too late to enhance oral care when individuals are elderly, or in a critical condition. Moreover, we cannot only pay attention to oral hygiene once tooth decay or periodontitis has occurred, since it is risky when the inflammation or infection affects the whole body system. Misperception of oral health status was associated with reducing in adopting oral hygiene habits, and decreasing the success of initiatives to address oral health-related diseases. Previous studies have identified subjective self-rated oral health status associated with objective clinical oral health condition [5,10]. Consequently, adequate oral hygiene behavior was limited. Theoretically, we should expect to maintain functional dentition throughout our lives. However, in previous studies, high prevalence of tooth loss, and poor adoption of oral hygiene behaviors have been observed in adults in rural areas [2,4]. Therefore, this study aimed to explore the subjective self-perception and objective condition of oral health among community adults in rural areas. The research finding will close the gap between incorrect understanding of oral health and its associated oral hygiene behaviors.

## 2. Materials and Methods

### 2.1. Study Design, Participants and Setting

This was a descriptive, cross-sectional, community-based study. The study was part of a nurse-led health promotion program, involving adults in rural communities, and a free health check-up performed by the cooperation local hospital in western coastal Taiwan. A sample size of 127 achieves 95% confidence in detecting an effect size of 0·35 using a 2-degree of freedom Chi-squared test with a significance level of 0·05. The actual sample size was 973, which is more than sufficient participants for a full statistical analysis.

### 2.2. Ethical Considerations

The study was approved by the institutional review board at the institution with which the authors were affiliated (IRB 103-6943B). We obtained the written informed consent from all participants after describing the study purpose and explaining that a free medical examination would be provided. A cover letter inviting individuals to participate in the study and stating that the responses would remain confidential was sent to community leaders.

### 2.3. Data Collection

Participants were recruited via convenience samples from December 2015 to July 2016. The inclusion criteria were as following: (1) age between 20–64 years, (2) full independence in performing activities of daily living, (3) ability to complete the questionnaires in Mandarin or a Taiwanese dialect during face-to-face interviews, (4) ability to understand and sign an informed consent form prior to enrollment in the study, and (5) ability to walk to the collaborating hospital. The exclusion criteria were (1) a serious learning difficulty, who demonstrates difficulty in learning about appropriate oral health practices, which may include those are disabled from a physical or intellectual disability (e.g., stroke or dementia), or (2) inability to complete the questionnaire. The survey instrument developed for the current study displayed good content validity index (CVI = 0.91–0.94) by seven experts (i.e., two dentists, two nursing faculty members teaching in health promotion, two endocrine physicians, and one cardiologist). We also invited three pilot participants to check the understanding of the instrument to examine face validity, and these participants were excluded from analysis. Some items within the instrument were revised, according to the suggestions provided by the experts and pilot participants. To assure the competency and reliability in delivering the research protocol, the primary investigator initiated four-hour training sessions, allowing all team members to familiarize themselves with the instruments. Ten research assistants were trained for 4 h by the investigators. Research assistants were senior nursing students and were divided into 5 pairs to interview each pilot participant and a 90% correct rate of inter-rater reliability was confirmed.

### 2.4. Measurements

#### 2.4.1. Self-Perception of Oral Health.

Participants were asked to rate their own oral health status by choosing one of five responses (very good, good, average, bad, or very bad) to the question, “How do you feel about/perceive your oral health”? These responses were categorized into two categories: good (very good, good, and average) and bad (bad and very bad).

#### 2.4.2. Oral Health Status 

This was measured by (a) number of remaining teeth (NRT), whereby a trained research assistant counted the natural and filled teeth, and (b) toothache/oral pain within the preceding 6 months, which was determined via the question, “Have you experienced toothache within the past 6 months, and if so, do you know the reasons for this oral discomfort”? Participants were asked to describe their most common reasons for visiting a dentist during this period.

#### 2.4.3. Oral Health-Related Behaviors

These were assessed by four domains representing eight oral hygiene habits: (1) Betel nut chewing/cigarette smoking (one question): “Do you chew betel nuts or smoke cigarettes”? Participants were classified as “nonusers” if they had never chewed betel nuts or smoked and “current or former users” if they currently or previously chewed betel nuts or smoked. (2) Personal oral hygiene (three questions): “How often do you brush your teeth after meal at least twice a day,” “how often do you use dental floss at least once every day” and “how often do you visit your dentist regularly (every 6 or 12 months) for check-ups and scaling”? The answers were categorized as “infrequently” (never/seldom) or “frequently” (usually/always). (3) Vegetable/fruit/water consumption habits (three questions): “How often do you eat three servings (one and a half bowls) of vegetables each day,” “how often do you eat two servings (one bowl) of fruit each day,” and “how often do you drink at least 1500 mL, or eight bowl-sized cups, of water (exclude beverages containing sugar) each day?” Responses were classified as “infrequently” (never/seldom) or “frequently” (usually/always). (4) Receiving oral hygiene education (one question): “According to your memory, have you received oral health/oral hygiene education from healthcare providers?” Responses were classified as “no” (never) or “yes” (at any time).

### 2.5. Data Analysis

Data are presented as numbers (percentages) for categorical variables and means (standard deviations) for continuous variables. Group comparisons (e.g., according to sex or perception of oral health) were performed using the chi-square test for categorical variables. Multivariate logistic regression analysis was performed to examine factors associated with perception of oral health as good or bad. The multivariate analysis was stratified according to sex to examine factors independently associated with perception of oral health. All variables were incorporated into the multivariate analyses and considered as confounders. The significance level was set at *p* < 0.05. All analyses were conducted using SPSS software version 17 (SPSS Inc., Chicago, IL, USA).

## 3. Results

### 3.1. Characteristics of the Participants

One thousand six (1006) community residents participated in the project, eventual 973 fulfilled the inclusion criteria and signed the consent form at the local hospital (Table 1). The mean age was 42.8 (SD = 12.3), and ranged from 20 to 64 years. Most participants (*n* = 512, 52.6%) were women, and more than half (64.8%) were educated to high school level or lower. The mean NRT was 25.2 (SD = 7.2); in addition, 153 participants (15.8%) had <20 remaining teeth, and 7.6% had ≤10 remaining teeth. Most of the participants (*n* = 702, 72.1%) reported brushing their teeth infrequently (never or seldom) after meals, 54.1% used dental floss infrequently, and 64% received dental check-ups or scaling infrequently. One third of participants consumed water (1500 mL/per day) infrequently, 31.2% consumed vegetables (three servings per day) infrequently, 40.3% consumed fruit (two servings per day) infrequently, and 27.4% were current users of cigarettes or betel nuts. Almost one third of participants (*n* = 287, 29.5%) had experienced toothache within the preceding 6 months; of these, 52.6% (*n* = 151) perceived their oral health as good. Two thirds of participants (66.9%) reported that the most common reason that they had visited a dentist during the preceding year was oral discomfort; of these, 61.4% perceived their oral health as good. More than half of the participants (*n* = 541, 55.6%) reported never having received oral hygiene education from healthcare providers.

### 3.2. Factors Associated with Self-Perception of Oral Health Status

Univariate analysis showed that participants who perceived their oral health as good were more likely to be younger (age ≤ 40, *p* < 0.001); be educated to a higher level (*p* < 0.001); have a higher NRT (*p* < 0.001); receive regular dental check-ups and scaling (*p* < 0.01); consume water (1500 mL/per day; *p* < 0.01), fruit, and vegetables frequently (*p* < 0.01); report greater satisfaction with their lives (*p* < 0.001); and have received oral hygiene education (*p* < 0.01) and less likely to have experienced toothache in the preceding 6 months (*p* < 0.001) or smoke or chew betel nuts (*p* = 0.001) relative to those who perceived their oral health as bad (Table 1). Table 2 shows the factors associated with having experienced toothache within the preceding 6 months, including having fewer remaining teeth (*p* < 0.05); undergoing regular dental check-ups and scaling infrequently; consuming water (1500 mL/per day; *p* < 0.01), vegetables (three portions per day; *p* < 0.01), and fruit (two portions per day; *p* < 0.05) infrequently.

After adjusting for confounding factors, multivariate logistic regression showed that a higher NRT (odds ratio (OR) = 3.03, 95% confidence interval (CI): 2.28–4.03), younger age (OR = 0.99, 95% CI: 0.98–0.99), regular dental check-ups/scaling (OR = 1.81, 95% CI: 1.31–2.40), higher levels of education (OR = 1.45, 95% CI: 1.03–2.04), and frequent water intake (OR = 1.38, 95% CI: 1.01–1.89) were independently associated with good self-perception of oral health status (Table 3).

In further analysis stratified according to sex, the results demonstrated that in women, a higher NRT (OR = 3.42, 95% CI: 2.32–5.04), younger age (OR = 0.98, 95% CI: 0.98–0.99), and regular dental check-ups/scaling (OR = 1.99, 95% CI: 1.33–2.99) were independently associated with perception oral health as good. In men, a higher NRT (OR = 2.86, 95% CI: 1.87–4.38), never having smoked or chewed betel nuts (OR = 0.51, 95% CI: 0.33–0.81), and without toothache within 6 months (OR = 1.02, 95% CI: 2.01–3.82) were independently associated with perception of oral health as good (Table 4).

## 4. Discussion

Four key findings regarding factors associated with self-perception of oral health in rural adults emerged from this study. First, there was a high prevalence of tooth loss and toothache in the study population. Second, many participants exhibited misperception of their oral health. Third, quite few participants had received oral hygiene education or practiced oral health-related behaviors. Fourth, with unmodifiable factors (e.g., sex, age, and educational level) adjusted for, a higher number of remaining teeth, and adopting oral health-related behaviors were significantly associated with self-perception of oral health as good.

The present findings showed that even participants had experienced toothache within the preceding 6 months; such as gingivitis, decay, or periodontitis, many of them still perceived their oral health as good. This phenomenon indicated that misperception of oral health would influence their objective clinical oral health condition and adopt inadequate oral hygiene behavior. For instance, in this study, many participants did not often brush teeth after meals, use dental floss, or regular dental check-ups, but reported their oral health condition as good. It is necessary for future study to close the gap between this mismatched for the community adults, particularly in the rural areas.

The results showed high prevalence of tooth loss and <20 remaining teeth in adults in rural areas. These findings are consistent with those of previous researches examining the prevalence of and factors associated with tooth loss in rural communities, which characterized with low educational levels, infrequent use of dental floss, and high prevalence of smoking [4,24,25]. The results also echoed the statements by WHO [1] that social determinants in oral health are very strong. The prevalence of oral diseases is increasing in all countries; the oral disease burden is significantly higher among poor and disadvantaged population groups. Tsai et al. [4] reported that 16.3% of rural adults had <20 remaining teeth and reported poor oral hygiene. Notably, 29.5% of participants in the present study had experienced toothache within the preceding 6 months. Further exploration showed that the reasons for participants’ toothache, as diagnosed by a dentist, included dental caries, periodontal disease, tooth loss, gingivitis, bleeding, purulence, gum swelling, and dental implants (not shown in the table). In addition, more than half of the participants reported that the most common reasons that they had visited a dentist during the preceding year were toothache or discomfort; however, 61.4% of these mentioned participants perceived their oral health as good. The phenomenon could have occurred because the participants had not been provided with an accurate definition of good dental health and perceived their oral health as good because tooth loss and toothache did not cause extensive disruption in their lives.

Good oral health is an asset not only at an individual level but also at a national level, particularly with respect to the burden exerted on national health insurance. Surprisingly, 55.6% of participants reported never having received oral hygiene education from healthcare providers. The 12 years of compulsory education in Taiwan has already included oral hygiene education for children, but similar education for adults is scarce, particularly in disadvantaged areas. Joanne Disch [26], the president of the American Academy of Nursing, stated that the importance of oral care in infection prevention has been well substantiated via research, and it should be a high priority for adults. However, this is not reflected in current research or organizational policy. Therefore, our findings strongly suggest that community nurses and dentists should collaborate to fill the gap of ideal perception of oral health through efficient oral health-related education. As most adults had entered the industrial sector, they had neglected to maintain their oral hygiene as they were taught to do at school. Or even worse, they didn’t ever physically receive the accurate oral hygiene education during their school days. These individuals could require only reminders and encouragement to practice oral hygiene.

Some studies have shown that individuals, who smoked and did not attend regular dental check-ups, nor brush their teeth, adhere to a balanced diet, nor consume vegetables or water, would frequently reported lower levels of satisfaction with life [1,24]. In addition, “8020 achievers” exhibited higher daily activity levels, greater satisfaction with life, and higher cumulative survival rates relative to those of “8020 non-achievers” [18]. This finding indirectly echoes the contribution that oral health makes to quality of life. For instance, regardless of sex, age, and educational level, participants’ perception of their oral health was correlated with their satisfaction with life, number of remaining teeth, and oral health-related behaviors. Therefore, the promotion of education regarding ideal oral health (e.g., via local media or radio) is a cost-effective strategy.

Oral health is essential to overall health and quality of life [1]; it plays an important role in the capacity for biting, chewing, smiling, and speaking, and enhances psychosocial wellbeing. The maintenance of good oral hygiene is an important strategy for the prevention of many chronic diseases. Research has shown that dental disease can be prevented easily in all age groups, through daily oral hygiene, adherence to a healthy diet, and avoidance of smoking [16]. However, the provision of oral hygiene-based care is discretionary and often omitted in hospital and community settings [4,15]. Further exploration of fundamental nursing care should be likely to frequently demonstrate the collaboration between nursing disciplines and address this issue. For instance, we should consider embedding basic oral hygiene care for each age group into the nursing curriculum and working with dentists in rural areas.

The study was subject to several limitations. First, because of the limited numbers of dentists in rural areas, participants’ oral health status was assessed by nursing assistants rather than dentists. Therefore, data regarding oral problems, such as toothache and discomfort were collected via self-report. This could have underestimated some oral problems, such as periodontal disease, and excluded dental implants. Second, the participants were not randomly recruited and were from the same geographic area, which limits the generalizability of these findings. Third, self-reports of personal health-related behaviors (e.g., vegetable consumption and cigarette smoking) could have been under- or overestimated because of recall bias.

## 5. Conclusions

Despite these limitations, the findings revealed a high prevalence of tooth loss, toothache, poor oral hygiene, and misperception of oral health in adults in rural areas. From the study findings, we suggest that oral health assessment and education for adults should be a routine part of all general health assessments in rural areas. It is necessary for primary health care providers to initiate oral health-related programs to reduce misperception, tooth loss, and toothache in rural areas. Further studies could reduce the consequences of misperception of oral diseases and oral health-related behaviors through the implementation of community-based oral health promotion programs.

## Figures and Tables

**Table 1:** Factors associated with perception of oral health status (N = 973).

Variables	Perception of Oral Health Status			
	Feel Bad	Feel Good		
	N (%)		*X* ^2^	*p*
Gender				
Female	158 (54.7)	354 (51.8)	0.69	0.405
Male	131 (45.3)	330 (48.2)		
Age (years)	Range 20–64 Mean (SD) = 42.8 (12.3)			
≤40	105 (36.3)	364 (53.2)	23.20	<0.001
≥41	184 (63.7)	320 (46.8)		
Educational level				
≤High school	224 (77.5)	407 (59.5)	28.90	<0.001
≥College and above	65 (22.5)	277 (40.5)		
Number of remaining teeth	Range 0–32 Mean (SD) = 25.2 (7.2)			
≤24	150 (51.9)	147 (21.5)	88.60	<0.001
≥25	139 (48.1)	537 (78.5)		
Brushing teeth after meal				
Infrequent (never/seldom)	218 (75.4)	484 (70.8)	2.21	0.137
Frequent (usually/always)	71 (24.6)	200 (29.2)		
Using dental floss every day				
Infrequent (never/seldom)	169 (58.5)	357 (52.2)	3.23	0.072
Frequent (usually/always)	120 (41.5)	327 (47.8)		
Regular dental check-ups/scaling				
Infrequent (never/seldom)	206 (71.3)	415 (60.7)	9.90	0.002
Frequent (usually/always)	83 (28.7)	269 (39.3)		
Water intake (1500 mL/per day)				
Infrequent (never/seldom)	115 (39.8)	208 (30.4)	8.07	0.005
Frequent (usually/always)	174 (60.2)	476 (69.6)		
Vegetable (3 servings)				
Infrequent (never/seldom)	110 (38.1)	194 (28.4)	8.90	0.003
Frequent (usually/always)	179 (61.9)	490 (71.6)		
Fruit (2 servings)				
Infrequent (never/seldom)	136 (47.1)	256 (37.4)	7.84	0.005
Frequent (usually/always)	153 (52.9)	428 (62.6)		
Smoking/betel nut chewing				
Current or former users	100 (34.6)	167 (24.4)	10.59	0.001
Non users	189 (65.4)	517 (75.6)		
Toothache within 6 month			60.97	<0.001
No	153 (52.9)	533 (77.9)		
Yes	136 (47.1)	151 (22.1)		
Reasons to visit dentist during the past 1 year			31.75	<0.001
Dental check-ups	51 (17.6)	239 (34.9)		
Oral discomfort	231 (79.9)	420 (61.4)		
Others (cosmetic)	7 (2.4)	25 (3.7)		
Receiving oral hygiene education				
No	180 (62.3)	361 (52.8)	7.44	0.006
Yes	109 (37.7)	323 (47.2)		

**Table 2:** Toothache within 6 month and health-related behaviors (N = 973).

Variables	Toothache within 6 Months			
	No	Yes		
	N (%)	N (%)	*X* ^2^	*p*
Number of remaining teeth				
≤24	196 (28.6)	101 (35.2)	4.18	0.041
≥25	490 (71.4)	186 (64.8)		
The reasons to visit dentist				
Dental check-ups/scaling	222 (32.4)	68 (23.7)	15.58	<0.001
Oral discomfort	435 (63.4)	216 (75.3)		
Others (cosmetic)	29 (4.2)	3 (1.0)		
Brushing teeth after meal/twice per day				
Infrequent (never/seldom)	500 (72.9)	202 (70.4)	0.63	0.427
Frequent (usually/always)	186 (27.1)	85 (29.6)		
Using dental floss every day				
Infrequent (never/seldom)	373 (54.4)	153 (53.3)	0.09	0.762
Frequent (usually/always)	313 (45.6)	134 (46.7)		
Regular dental check-ups (1/2-1 year)				
Infrequent (never/seldom)	447 (65.2)	174 (60.6)	1.80	0.180
Frequent (usually/always)	239 (34.8)	113 (39.4)		
Receiving oral hygiene education				
No	386 (56.3)	155 (54.0)	0.42	0.517
Yes	300 (43.7)	132 (46.0)		
Water intake (1500 mL/per day)				
Infrequent	207 (30.2)	116 (40.4)	9.57	0.002
Frequent	479 (69.8)	171 (59.6)		
Vegetable (3 servings/per day)				
Infrequent (never/seldom r)	197 (28.7)	107 (37.3)	6.91	0.009
Frequent (usually/always)	489 (71.3)	180 (62.7)		
Fruit (2 servings/per day)				
Infrequent (never/seldom)	260 (37.9)	132 (46.0)	5.51	0.019
Frequent (usually/always)	426 (62.1)	155 (54.0)		
Smoking/betel nut chewing				
Non users	504 (73.5)	202 (70.4)	0.96	0.325
Current users or formerly	182 (26.5)	85 (29.6)		

**Table 3:** Factors associated with perception good of oral health status.

Variables	B	S.E.	Odds Ratio	*p*	95% CI *
Number of remaining teeth (1 ≥ 25)	1.11	0.15	3.03	<0.001	2.28–4.03
Age (per year)	−0.01	0.01	0.99	<0.001	0.98–0.99
Dental check-ups/scaling (1 = Regular)	0.61	0.13	1.81	<0.001	1.31–2.410
Educational level (1 = high)	0.37	0.18	1.45	0.035	1.03–2.04
Water intake 1500 mL/day (1 = often)	0.32	0.16	1.38	0.047	1.01–1.89

* CI: confidence interval.

**Table 4:** Factors associated with perception good of oral health status stratified by gender.

Variables	B	S.E.	Odds Ratio	*p*	95% CI *
Female					
Number of remaining teeth (1 ≥ 25)	1.23	0.20	3.42	<0.001	2.32–5.04
Age (per year)	−0.02	0.01	0.98	0.002	0.98–0.99
Dental check-ups/scaling (1 = Regular)	0.69	0.19	1.99	0.001	1.33–2.99
Male					
Number of remaining teeth (1 ≥ 25)	1.05	0.22	2.86	<0.001	1.87–4.38
Smoking/betel nut chewing (1 = yes)	−0.67	0.23	0.51	0.004	0.33–0.81
Toothache within 6 month (1 = no)	1.02	0.16	2.77	<0.001	2.02–3.82

* CI: confidence interval.
